# Fourier transform infrared spectroscopic imaging of colon tissues: evaluating the significance of amide I and C–H stretching bands in diagnostic applications with machine learning

**DOI:** 10.1007/s00216-019-02069-6

**Published:** 2019-08-16

**Authors:** Cai Li Song, Martha Z. Vardaki, Robert D. Goldin, Sergei G. Kazarian

**Affiliations:** 1 Department of Chemical Engineering, South Kensington Campus, Imperial College London, London, SW7 2AZ UK; 2 Michael Smith Laboratories, The University of British Columbia, Vancouver, BC V6T 1Z4 Canada; 3 Department of Cellular Pathology, St. Mary’s Campus, Imperial College London, W2 1NY, London, UK

**Keywords:** Fourier transform infrared spectroscopic imaging, Colon polyps and cancer, Correcting lens approach, Machine learning, K-means clustering, Random forest supervised classification

## Abstract

Fourier transform infrared (FTIR) spectroscopic imaging of colon biopsy tissues in transmission combined with machine learning for the classification of different stages of colon malignancy was carried out in this study. Two different approaches, an optical and a computational one, were applied for the elimination of the scattering background during the measurements and compared with the results of the machine learning model without correction for the scattering. Several different data processing pathways were implemented in order to obtain a high accuracy of the prediction model. This study demonstrates, for the first time, that C–H stretching and amide I bands are of little to no significance in the classification of the colon malignancy, based on the Gini importance values by random forest (RF). The best prediction outcome is found when supervised RF classification was carried out in the fingerprint region of the spectral data between 1500 and 1000 cm^−1^ (excluding the contribution of amide I and II bands). An overall prediction accuracy higher than 90% is achieved through the RF. The results also show that dysplastic and hyperplastic tissues are well distinguished. This leads to the insight that the important differences between hyperplastic and dysplastic colon tissues lie within the fingerprint region of FTIR spectra. In this study, computational correction performed better than optical correction, but the findings show that the disease states of colon biopsies can be distinguished effectively without elimination of Mie scattering effect.

**Electronic supplementary material:**

The online version of this article (10.1007/s00216-019-02069-6) contains supplementary material, which is available to authorized users.

**Figure:**
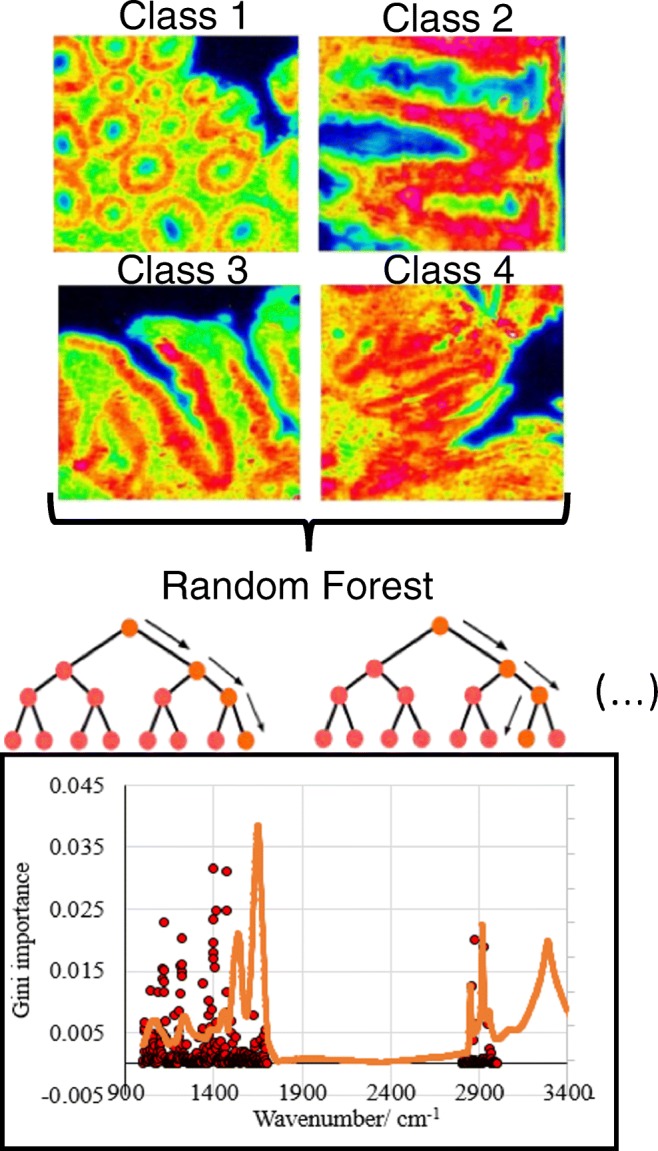
Graphical abstract

## Introduction

Colon cancer is a disease in the large intestine in which abnormal cells divide uncontrollably. Most cases of the colon cancer begin as a small adenomatous polyp which lines the inner surface of the colon [1]. In the UK, colon cancer is the fourth most common cancer with 16,000 deaths every year, making it the second most common cause of cancer death in 2016 [2]. Early detection of colon cancer can help reduce mortality and morbidity. The current diagnostic approach for this disease includes biopsy collection followed by histopathology during colonoscopy or surgery. In recent years, FTIR spectroscopy has been shown as a promising technique to enhance the clinical diagnosis in a label-free way by investigating the chemical content of the biopsy samples [3–6].

Although FTIR spectroscopy has the potential as a clinical prognostic tool, there are several challenges associated with it, most notably the reflection and scattering contribution (‘dispersion artefact’) that arise from the spatial inhomogeneity of the sample. In fact, the dispersion artefact is found to be largely dominated by resonant Mie scattering in pure transmission experiment, as opposed to measurement in transflection mode where the reflection artefact becomes significant [7]. The scattering contribution can lead to spectral distortion, for example, a decrease in the absorbance of the amide I band, and to a greater extent, manifest itself as a derivative-like baseline at the high wavenumber side of the amide I band. It can also result in significant frequency shifting of spectral bands that are used extensively to classify biological specimens. To be able to interpret FTIR spectra correctly, it requires the correction of the dispersion artefact aforementioned. The origin of dispersion artefact and their subsequent effect is detailed in published articles [7, 8].

Resonant Mie scattering (RMieS) algorithm developed by Bassan et al. was proved to be successful at correcting the ‘dispersion arterfact’ [7]. This algorithm is used in this manuscript; however, the correction algorithm is computationally intensive and time consuming. In addition, physical alteration of imaging set-up for measurements in transmission by employing an additional lens on top of the window that forms pseudo-hemisphere has shown to be effective at producing aberration-free and high-quality spectra from tissues and from cells [9–12]. The other challenge in FTIR spectroscopic measurements is the presence of spectral bands of water vapour in the sample spectra, which hampers the analysis of protein secondary structure in the amide I region (1700–1600 cm^−1^) [13–15]. This water vapour interference can be minimised by computational subtraction of the pure water vapour spectrum from the sample spectrum, with algorithm described by Brunn et al. [16].

FTIR spectra contain a wealth of information about the sample. As such, in analysis of spectra of biological systems, multivariate statistics and machine learning algorithms are frequently applied to extract the important information. The two main strategies in chemometrics used to analyse FTIR spectral data are unsupervised learning and supervised learning. The variety of the methods is detailed by Goodacre [17]. The aim of this paper is to utilise the well-established machine learning approach, random forest in this case, to examine the spectral ranges that could potentially contain the most important spectral biomarkers that distinguish between colon specimens of various degree of malignancy.

## Materials and methods

### Sample preparation

The samples are formalin-fixed paraffin-embedded (FFPE) colon biopsies at different disease stages of malignancy (hyperplasia, dysplasia, and cancer), provided by St. Mary’s Hospital (Imperial College London, UK), following standard clinical protocols. The samples were microtomed at 3 μm thickness from a specimen block and mounted onto a 2-mm-thick CaF_2_ window (Crystran Ltd., UK) for FTIR analysis. The adjacent section was mounted onto a glass slide, stained with haematoxylin and eosin (H&E) and assessed by a trained pathologist. The sections deposited on CaF_2_ windows were deparaffinised as per the procedure described by Song et al. [18, 19] and stored with a desiccant before use.

### FTIR spectroscopic imaging measurements

The experiments were carried out in transmission mode at × 15 magnification (NA = 0.4), with a Hyperion 3000 FTIR microscope coupled to Tensor 27 FTIR spectrometer (Bruker Corp.). A liquid nitrogen cooled 64 × 64-pixel focal plane array (FPA), which has a field of view of 170 × 170 μm^2^, is used for simultaneous acquisition of FTIR spectral dataset. As imaging was combined with mapping, 3 × 3 individual images were stitched into one, resulting in a total measured area of 510 × 510 μm^2^ for each tissue. The spectral images from 12 sample areas were acquired. A new background was recorded before measuring each individual image. All measurements were taken in the mid-IR range from 3900 to 900 cm^−1^, at 4 cm^−1^ spectral resolution and with 521 co-added scans. An additional CaF_2_ lens, which has been shown to significantly reduce Mie scattering [12], was also employed for imaging of the exact same tissue areas. The design and set-up of the lens for combining imaging with mapping were described in details by Kimber et al. [20]. To put it briefly, the added lens is kept in focus with an external holder whilst the stage is shifted in x- and y-direction for different areas to be measured.

The additional lens implemented to correct for the chromatic aberration in infrared measurement is referred to as ‘correcting lens’ from this point onwards in this paper. To the authors’ knowledge, the assessment of the performance of the correcting lens has not been closely examined with advanced machine learning approaches.

### Data processing and chemometric analytical procedure

The spectral data were processed with MATLAB R2018b (The MathWorks, Inc.). The spectral data in the range of 1800–1000 cm^−1^ and 3000–2800 cm^−1^ were used for further analysis. The region between 2800 and 1800 cm^−1^ contains no important spectral information whilst the region > 3000 cm^−1^ is sensitive to water content within the tissues. Baseline correction and water vapour subtraction were not applied to the data. Second derivatives of the obtained spectra were calculated with Savitzky-Golay 9-point smoothing, which were then vector normalised. The spectra, second derivatives, and normalised second derivative data were then separately subjected to unsupervised machine learning, in this instance, the K-means clustering algorithm (tested for 2 to 6 clusters, each with 5 replicates and infinite iteration until the solution converges to a local minimum). A total of 2000 individual sample spectra were recorded and used for machine learning. Only eight chemical images of the different tissue sections are shown here for demonstration purpose. Training and test models were created, each made up of 500 random spectra sampled from each cluster without replacement, for tissue at the same disease stage. In other words, the model consists of 2000 spectral data (500 for healthy (H), 500 for hyperplastic polyps (HY), 500 for dysplastic polyps (D), and 500 for cancer sections (C)) which are identified by H&E staining. The models were from different individuals ensuring that the inter-patient variability is included in the study. Employing machine learning to study imaging data has been demonstrated in previous works [17, 21].

**Fig. 1:**
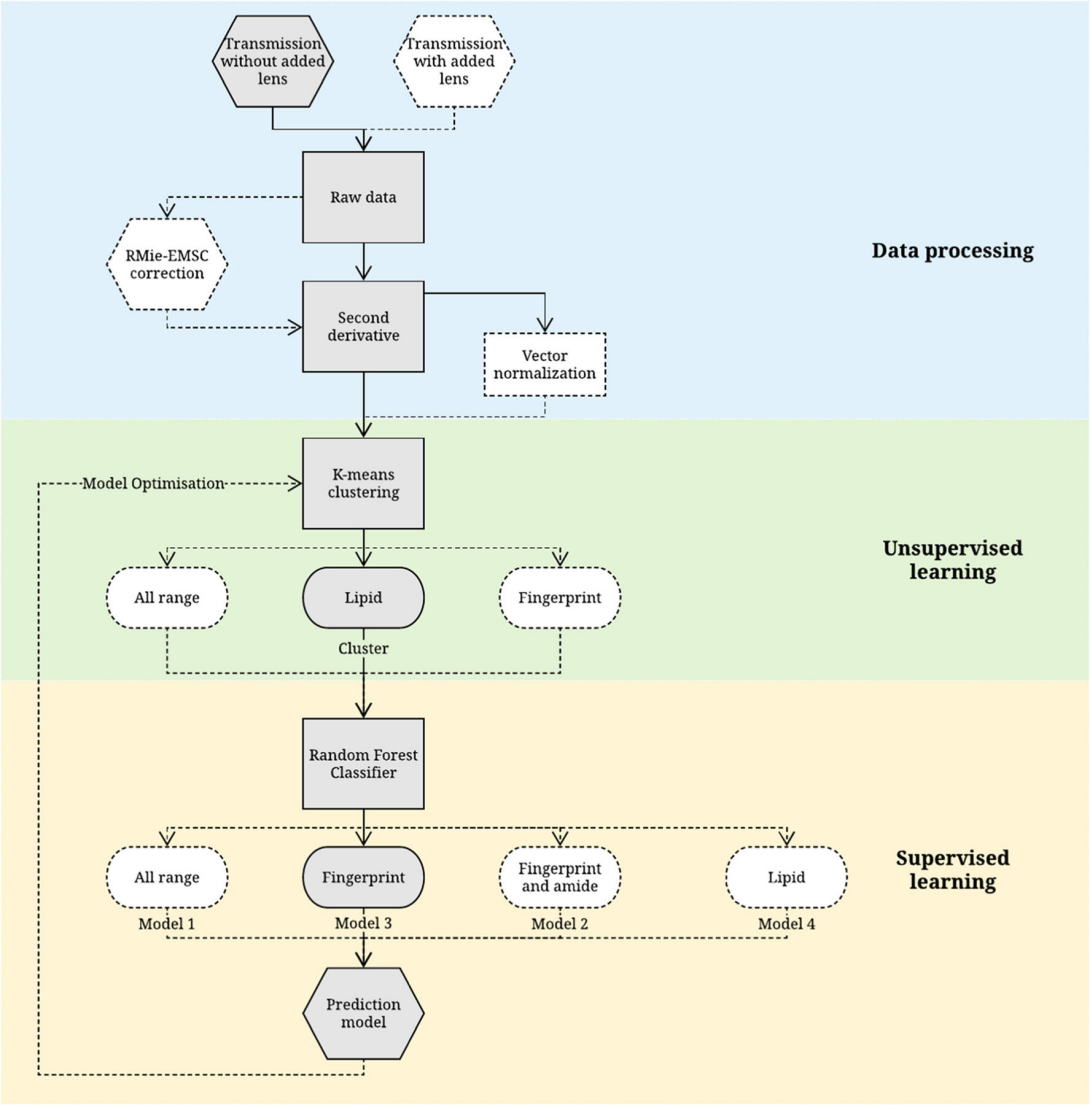
Schematic overview of the data processing and machine learning steps explored in this study. The best pathway leading to the optimised result is highlighted in grey

The training model, after undergoing data dimensionality reduction with PCA, was subsequently supplied to random forest (RF) classifier to generate a prediction model on the test model. RF operates by constructing multiple decision trees for classification on the data, gets prediction from each tree and thus outputs the class mode by means of voting. In this study, bootstrapping as well as a five-fold cross validation of the dataset is implemented [22]. Among various supervised machine learning classifiers, RF is preferable since it is faster and insensitive to over-fitting [23]. The prediction accuracy of the RF model is presented in the form of a confusion matrix. Inter-model predictability was carried out with independent training and test set. The size of training to test models was varied from 1:1 to 1:6. The analytical procedure is repeated with the measurement data obtained from the added correcting lens, as well as for no-lens data but corrected with RMieS algorithm (provided by Peter Gardner’s Lab, University of Manchester) [7, 24–26]. The parameters of the RMieS algorithm are given in Electronic Supplementary Material (ESM) (Table S1). Several machine learning parameters, namely the number of clusters, the spectral range for supervised and unsupervised classification, the size of training and test models, and the variance of retained PCA, have been tried and tested, to optimise the prediction model. The important features are selected from the Gini index—a score of the feature importance that is derived from the training of the RF classifier, which technically correlates to the optimal ‘Gini impurity’ split at each nodes within the binary trees [27]. Based on the selected features or spectral range, a flowsheet depicting all the different pathways to re-training machine leaning in categorising the different stages of the colon cancer is shown in Fig. 1. The prediction accuracy of the test models was used as the criteria to cross-check the spectral range highlighted by the machine learning as the ‘key biomarker’ that can be utilised to understand different degree of malignancy of colon.

Figure 1 shows all the pathways that are tested with unsupervised and supervised approach via training and re-testing of the spectral data based on Gini importance (see figure in section ‘Gini index obtained from RF classifier’). The features in this study are not independent of one another; thus, important spectral range is discussed instead of the single features. The paper starts off by describing Mie scattering and the implementation of correcting lens on the FTIR spectra of colon tissue, followed by the unsupervised learning on the second derivative spectra without RMieS correction. With supervised classifier, the performance of both model with and without any RMieS correction is compared and discussed to establish a proof of concept that RMieS correction might not be necessary in this case study. On top of that, this paper serves to demonstrate that amide I band plays a very little role in the differences between specimens via feature selection in machine learning. The results are detailed as follows.

## Results and discussion

### Physical and computational correction of Mie scattering effect

**Fig. 2:**
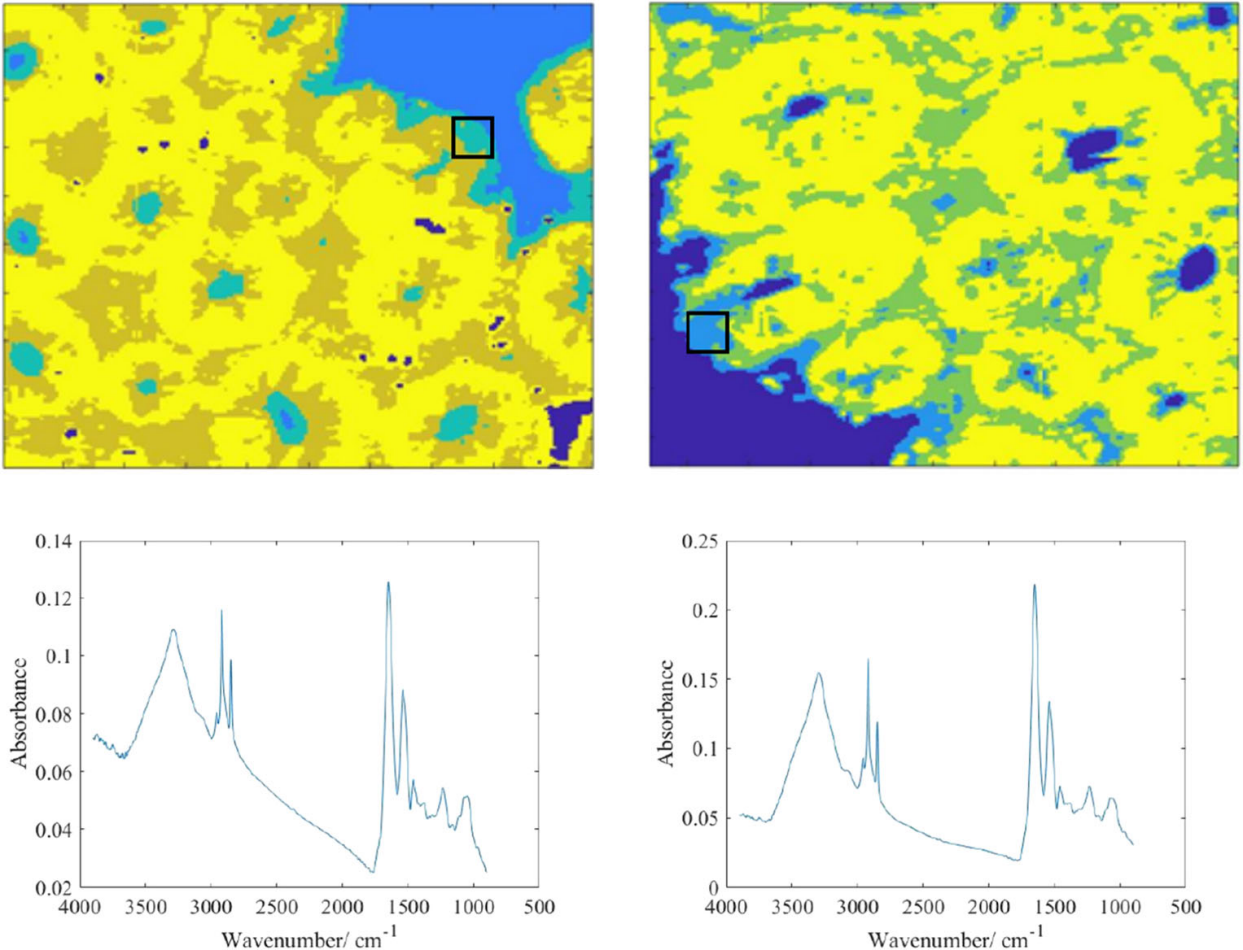
Top: false colour K-means cluster images of healthy colon tissue without the lens (left) and with the lens (right) obtained by mapping from nine stitched images. Each of the chemical images has a size of 510 × 510 μm^2^. Cluster represented in light blue shade (box) indicates the edges of the tissue. Bottom: the average measured spectra from the areas representing the edges of the tissue

**Fig. 3:**
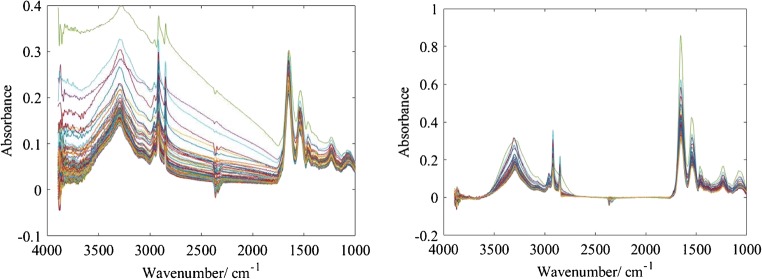
The raw spectra of 100 random pixels before and after RMieS correction, shown on the left and on the right respectively. Severe Mie scattering effect can be seen in the figure on the left prior to correction

Mie scattering effect is significantly reduced at the edges of the tissues when correcting lens is added, shown in Fig. 2, where increase in absorbance of the amide I band and reduction in the sharp derivative-like distortion to the spectra at ~ 1710 cm^−1^ are observed. Correction with added lens slightly shifted the peak position of the amide I band by ~ 1 cm^−1^ from 1652 to 1653 cm^−1^. With the added lens, which acts like an immersion objective as reported by Kimber et al. [20], the image has ~ 40% increase in magnification (total area of 360 × 360 μm^2^ compared with 510 × 510 μm^2^ for image without lens) and is flipped due to the arrangement of the tissue during measurement whereby the tissue is placed facing downwards with the correcting lens added on top of it. Computation correction with RMieS algorithm was more efficient at recovering a flat baseline of the spectra (Fig. 3) compared with correction with the added lens but was more time consuming. The peak position of amide I band was corrected to where the peak is supposed to be at 1654 cm^−1^ with the computational method.

### Data processing

**Fig. 4:**
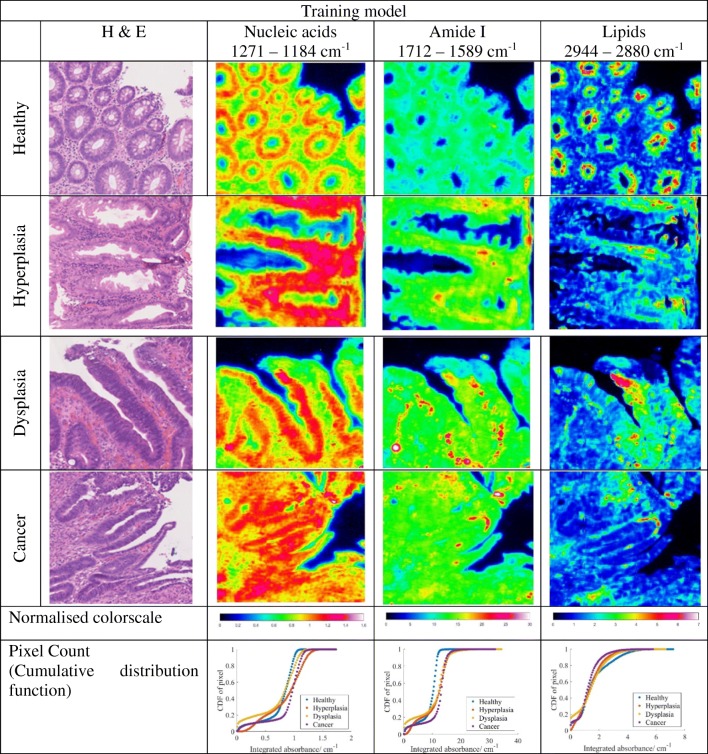

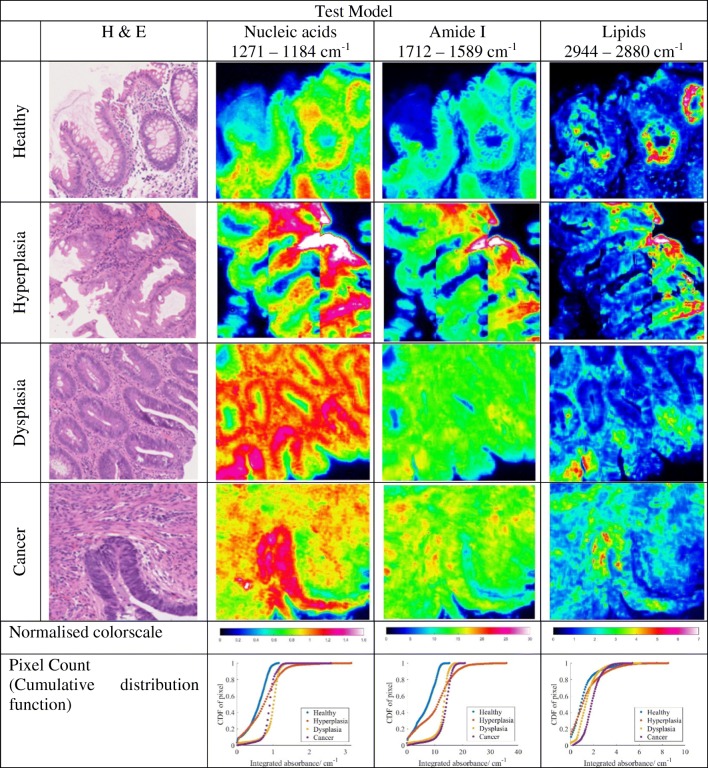
FTIR spectroscopic images of the colon biopsy used in the training models (top) and test models (bottom), depicting the distribution of different components by evaluating the integrated absorbance at various spectral ranges, which are labelled at the top of each column. The first column gives the H&E-stained images identified by the pathologist. Each image has a size of 510 × 510 μm^2^.The colorscale of the images is normalised across each component (column-wise) for comparison of their absorbances.

Eight different tissue regions, which comprise of 4 training and 4 test models (as described in data processing procedure), are measured and analysed. The number of areas taken in total is 12 (four disease states for three patients). Chemical images showing the distribution of integrated absorbance, estimated with trapezoidal rule of integration, at the spectral bands of 1272–1184 cm^−1^, 1712–1589 cm^−1^, and 2944–2880 cm^−1^, which are assigned to asymmetric phosphate stretching of nucleic acid, amide I, and CH stretching of lipid respectively [28], are represented in Fig. 4 (top: training model; bottom: test model; only eight samples were selected for comparison here), alongside the H&E stain images, which were used by pathologists to assign the stage of tissue malignancy. The cumulative distribution curves of the pixel count of each integrated absorbance (with the data sorted into 100 bins) are also shown for easier comparison of the chemical images. Although the pixel count is by no means an accurate indicator of the specific variation between disease stages as it is dependent on the area of measurement, it provides a quantitative means for direct comparison of the chemical images which is not quite so distinct even after normalisation of the colour scale.

As can be seen in the curves of pixel count Fig. 4, the integrated absorbance of nucleic acid band at 1271–1184 cm^−1^ is lowest for healthy colon biopsy when a 95% confidence interval is taken, likewise for amide I band. The opposite is observed for the lipid spectral band within 2944–2880 cm^−1^, whereby the lowest integrated absorbance is achieved in cancer tissues. This is in agreement with the high nucleic acid-to-cytoplasmic ratio observed in colon cancer tissues [29] as well as the loss of normal glandular architecture. The inner lining or mucosa of healthy colon is lined with columnar epithelium and large number of goblet cells, where numerous secretory vesicles containing mucus (glycoprotein) are present, in addition to the secreted mucin in the intestinal epithelial surface layer. Mucus is a complex biochemical layer made up of carbohydrates, antimicrobial peptides, immunoglobulins, electrolytes, and lipids [30]. For diseased tissue, however, the goblet cells are not differentiated well to perform its function; instead, they become highly metastasizing cells with high metabolic rate, which might progress to cancer (an aggregation of undifferentiated cells).

The difference between different stages of cancer is also highlighted in the mean average spectrum obtained after taking their second derivatives. The evaluation of the variation is not very straightforward, thus the need for machine learning to perform the task of classification of colon disease. The interpretation of the second derivative spectra is not included in the main discussion as machine learning only requires the input of ‘features’, which is the absorbance at various wavenumbers, and ‘label’, the stage of disease. The second derivative spectral bands and their corresponding band assignment are, nonetheless, provided in ESM Fig. S4 and Table S2 to demonstrate the potential variation that might be picked up by the machine learning classification model. The most significant differences lie in the peak shift and the intensity of the trough of the second derivative data.

### Gini index obtained from RF classifier

**Fig. 5:**
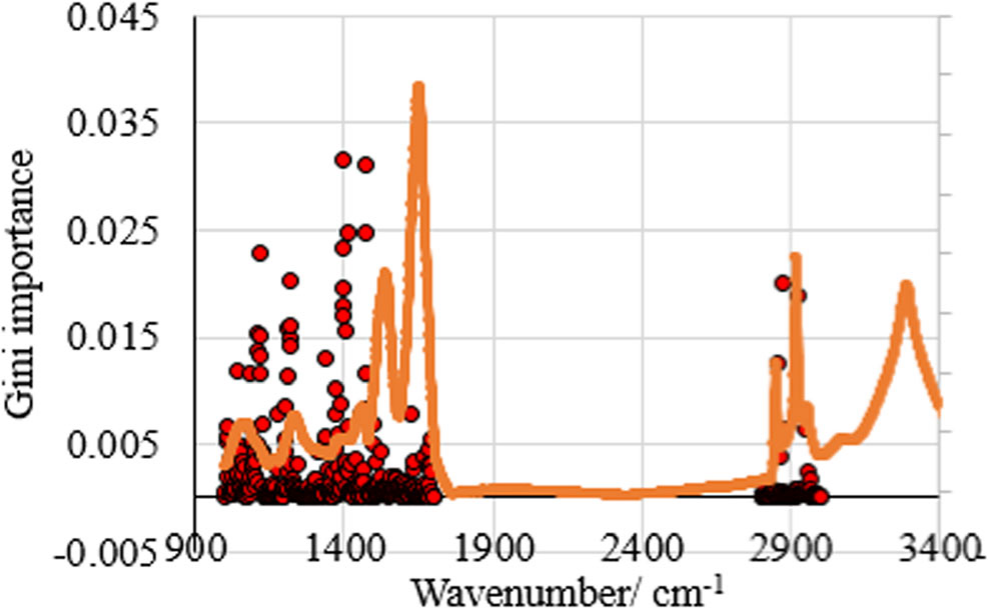
Plot of Gini importance values obtained from RF prediction model against wavenumber of colon biopsy tissue, overlaid on the average FTIR spectrum of healthy colon tissues (normalised between 0 and 0.040) for clarification purpose

The choice of spectral wavenumbers for classification, as mentioned earlier, is based on the Gini index (Fig. 5). The results show that the fingerprint region (< 1500 cm^−1^) contains the most important features (greater than Gini index of 0.015). This was followed by the lipid region (3000–2800 cm^−1^) of secondary importance. Surprisingly, the best prediction accuracy is obtained when unsupervised training is applied on the spectral range of secondary importance, whilst the most important features are used for supervised training. A similar machine learning study was performed by Kuepper et al. on colon cancer; however, the spectral range used for the training is inclusive of the amide I band [31]. It should be realised that the amide I region shows no significant importance, based on the Gini values demonstrated here.

### Unsupervised learning

**Fig. 6:**
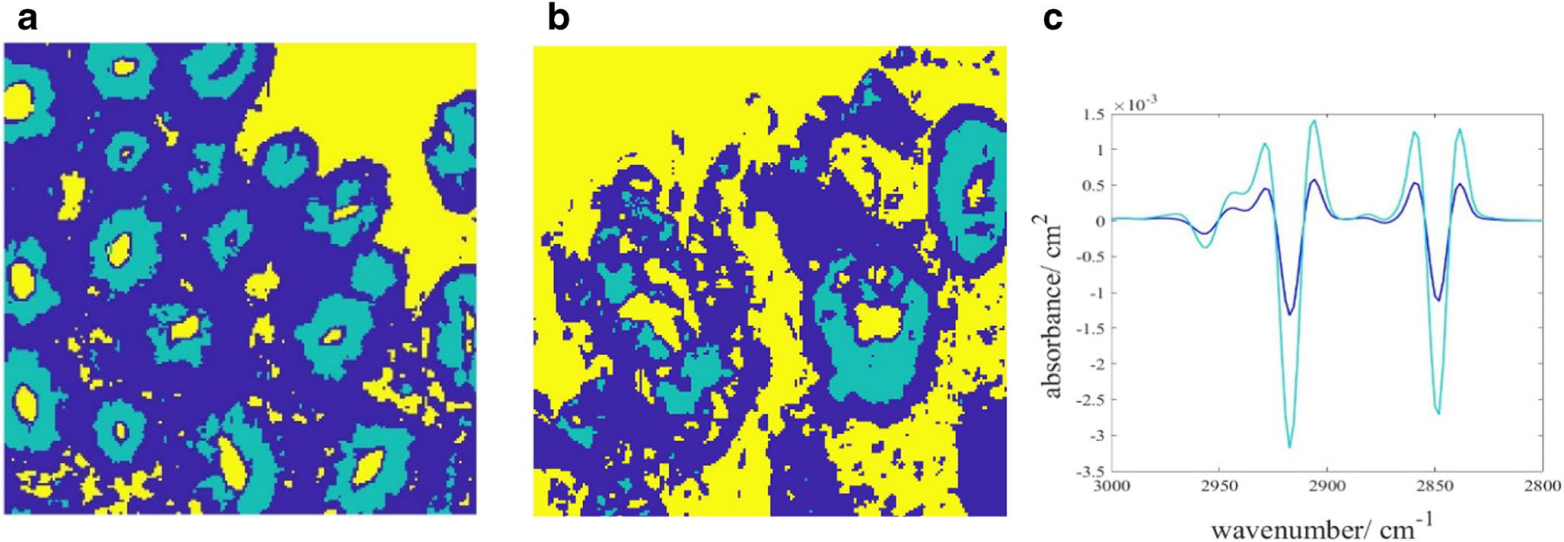
Representative colour-coded K-means clustered images of healthy colon biopsy sections of **a** test and **b** training model. Cluster represented in light blue is for areas dominated by goblet cells (denoted as cluster 2), dark blue for basal membrane (denoted as cluster 1), and yellow for areas without tissue. **c** Average second derivative spectra of the corresponding clusters in the high wavenumber spectral region (3000–2800 cm^−1^), following the colour code in K-means cluster

K-means clustering are used for intra-tissue classification, by maximising inter-distance variance between data within a tissue. It is important to recognise here that the optimum parameters for clustering in this study, after assessing the outcome of the supervised predictive model by comparing different spectral ranges and the number of clusters (results not shown), are based on the second derivative of the spectra between 3000 and 2800 cm^−1^ (introduced as the ‘lipid region’ henceforth, although strictly speaking, the spectral band within this region is not limited to lipid; it is assigned to the C-H stretching of methyl and methylene groups) [28]. The three clusters identified are considered sufficient in this study following these reasoning: first of all, the various tissue morphology categorised by the clusters are fed into supervised machine learning independently as a way of phasing out unnecessary regions of the tissue since not all morphology or clusters show essentially distinct spectra between different stages of colon disease; the highest performance is obtained with the spectra from high lipid region, which can be easily classified with just 3 clusters. Secondly, the higher the number of unsupervised clusters implemented, the higher the degree of similarity of the spectral data within each cluster; the lower the tolerance for dissimilarity of the test datasets, in other words, overfitting of data is introduced. In addition, since K-means is an unsupervised imaging approach, higher number of clusters have a tendency to cluster data that are close to each other which should have been treated as one (the sum of squared distances between each cluster decreases exponentially with increasing number of clusters). Besides, the main objective of this study is to assess the importance of lipid and amide bands in the prediction ability of the RF machine learning, the least number of clusters which can output a good predictive performance is desirable, in this case, three clusters for the intra-tissue differentiation can warrant a prediction outcome greater than 90% accuracy. Most importantly, higher category of classification (> 3 groups) is not needed as the lipid region is of secondary importance, as discussed before. Although the higher number of clusters are useful at exploration of the various histopathological architecture of colon adenocarcinoma, as analysed by Lasch et al. using similar multivariate imaging approaches [32], the tissue morphology is not explored in this paper. The false colour images generated from K-means clustering and their corresponding second derivatives are shown in Fig. 6.

From the mean second derivative spectra by averaging all the pixels within the same cluster, the tissue regions are effectively classified into low and high lipid absorbance region (cluster 1 and cluster 2 respectively), which are fed into the supervised learning algorithm separately. This reinforces the findings by Song et al. [18] that spectral bands of lipid are still useful biomarkers for intra-tissue classification, despite the lower Gini importance index. The lipid spectral region contains a wealth of information. Bassan et al. has also demonstrated that the high wavenumber spectral range (O−H, N−H, and C−H stretches occurring at ca. 3800–2500 cm^−1^) is useful for the generation of false colour classification image of breast tissue microarrays on glass substrate [33]. They are free from interference with the spectral bands of water vapour and Mie scattering, with the only possible variation coming from the deparaffinisation process on the formalin-fixed tissues. This variation is controlled and minimised by strictly adhering to the deparaffinisation protocol.

It is possible that the cancerous tissues are more susceptible to change during solvent-based removal of material, required prior to the paraffin embedding process. The FFPE process requires fixation of fresh tissue in formalin for 6 to 24 h, followed by multiple washes in ethanol/water with increasing ethanol concentration until water has been removed. Xylene, or possibly isopropanol, is then used to remove the ethanol, taking with it much of the fats within the natural tissue. Finally, the tissue is soaked in molten paraffin, usually at 60 °C. Precautions were taken to conduct the de-waxing process in a closely controlled manner, so that each of the three samples were treated in the same way; however, the manner in which the FFPE was first conducted is out of our control, including the amount of fats and other materials that may have been removed in that process. That said, surprisingly, similar observations were made on prostate cancer tissues that are supplied by different pathologists but de-waxed with the same procedure [18] that this wavenumber region (3000–2800 cm^−1^) is different between normal and cancer samples. Thus, the explanation that tissues of different malignancy retain various amount of fats after deparaffinisation essentially still offers a different kind of ‘key biomarker’ for cancer differentiation in FTIR imaging study.

### Supervised machine learning

Random forest classifier was shown to be an efficient supervised machine learning technique for the classification of spectral data in previous studies [17, 34, 35]. In this study, second derivative data (for measurements with and without correcting lens) from various spectral ranges were used to train the algorithm—model 1, between 1800 and 1000 cm^−1^ and 3000–2800 cm^−1^ (all range); model 2, 1800–1000 cm^−1^ only (fingerprint region with amide bands); model 3, 1500–1000 cm^−1^ only (fingerprint region); and model 4, 3000–2800 cm^−1^ only (lipid region). To clarify, re-training and re-testing of the RF models is still required after Gini selection to subjectively assess the prediction performance; hence, the results are organised in the way shown in the workflow (Fig. 1) in this manuscript.

**Fig. 7:**
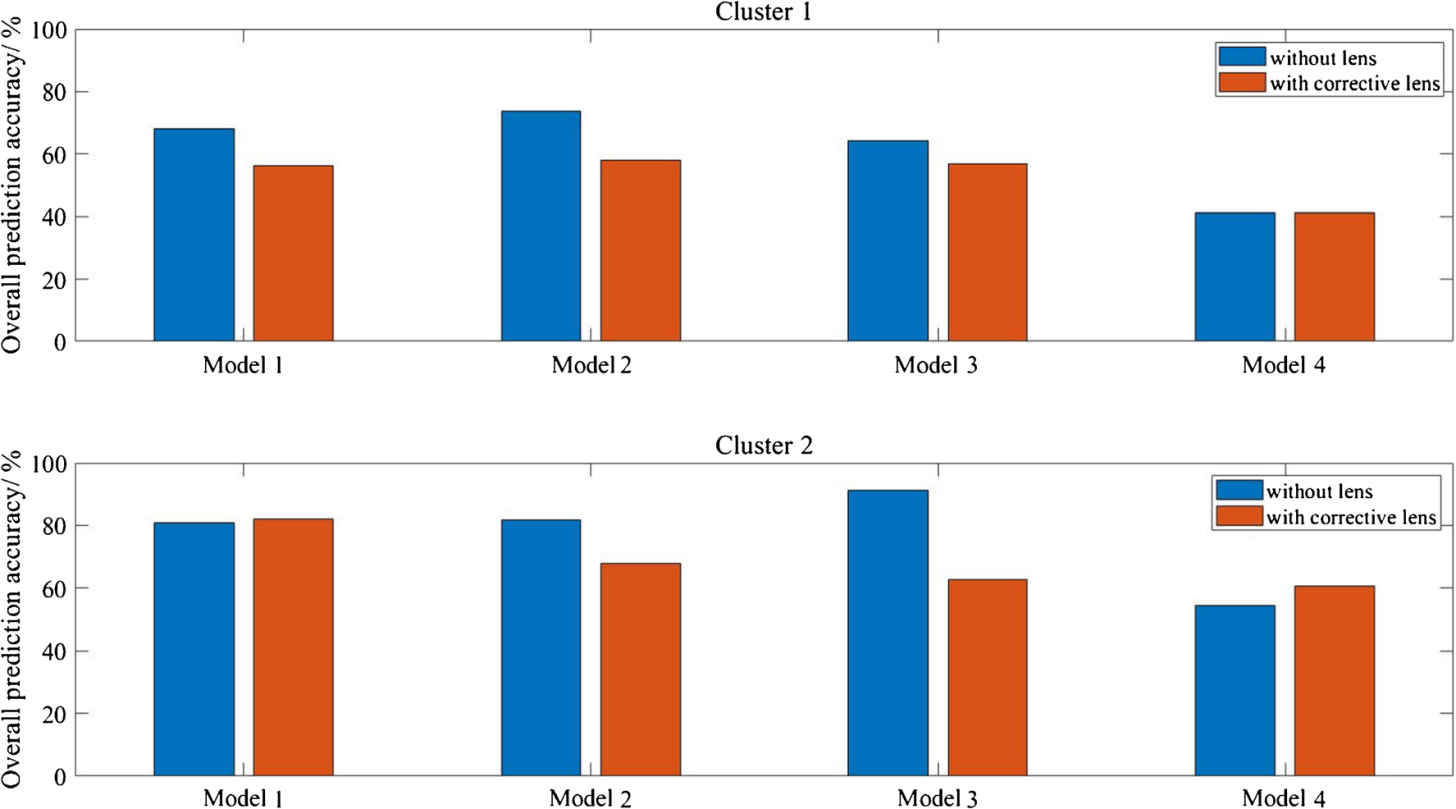
The bar chart shows the overall prediction accuracy in percentage of various models for measurement with and without correcting lens (and without computational correction for Mie scattering effect) for cluster 1 of low lipid absorbance and cluster 2 of high lipid absorbance

The overall prediction accuracy for each model is shown in Fig. 7. A typical fingerprint region of infrared measurement is loosely defined to be between ~ 1600 and 1500 cm^−1^ to 500 cm^−1^ [13, 36]. To avoid confusion to readers, in this paper, the fingerprint region is referring to spectral range within 1500–1000 cm^−1^ inclusive. At this part of the analysis, no computational correction of Mie scattering is applied.

Figure 7 shows that overall prediction accuracy is higher for data in cluster 2, region of higher lipid absorbance, than cluster 1. A comparison of the performance of measurements with and without correcting lens can be achieved by analysing cluster 2, which reveals that apart from model 1 and model 4, the measurements with correcting lens, despite its ability to minimise Mie scattering at the edges of the tissues, generally underperform compared with measurements without the added lens. The lowest accuracy of cluster 2 prediction is obtained from model 3 with added lens. This is because whilst the added lens approach removes the scattering effect and thus improves the quality of amide I band, the spectra collected in the range of 1100–1000 cm^−1^ suffer from enhanced noise, which is not an issue with computational approach. This happens because the additional stacking of lens on top of the CaF_2_ substrate (from the way the correcting lens is set-up) reduces the throughput of light. Due to the lower photon counts that pass through the sample and the fact that CaF_2_ has a cut-off at ~ 900 cm^−1^ in transmission, the spectral quality in the low wavenumber region deteriorates significantly compared with the set-up without correcting lens.

Model 2 (with lens) gives a slightly better performance when amide bands are factored into consideration as added lens is shown to improve the absorbance of the spectral band of amide I. Model 4 which considers the data exclusively from the lipid region is undeterred by the noise introduced by the extra lens configuration and model 1 which takes into consideration all the spectral regions shows similar performance with and without additional lens, for reasons discussed above. Instead of CaF_2_, a pseudo-hemispherical ZnS lens with infrared cut-off at ~ 700 cm^−1^ was suggested to improve the spectral quality [20]. However, Mie scattering correction does not play a significant role in optimising the performance of the supervised learning, reinforced by the finding that the highest prediction accuracy of 92.7% can be achieved with model 3 (fingerprint region). In other words, the second derivative spectral data within 1500–1000 cm^−1^ from cluster of high lipid absorbance region alone is sufficient to achieve effective discrimination of all the different grades of colon cancer as the fingerprint region is least affected by Mie scattering. The interference of the water vapour spectra within this region is also minimal (as shown in ESM Fig. S5). Therefore, removal of Mie scattering effect is not necessary as the amide spectral range (1700–1500 cm^−1^) does not need to be included in data analysis at all, as demonstrated here.

**Fig. 8:**
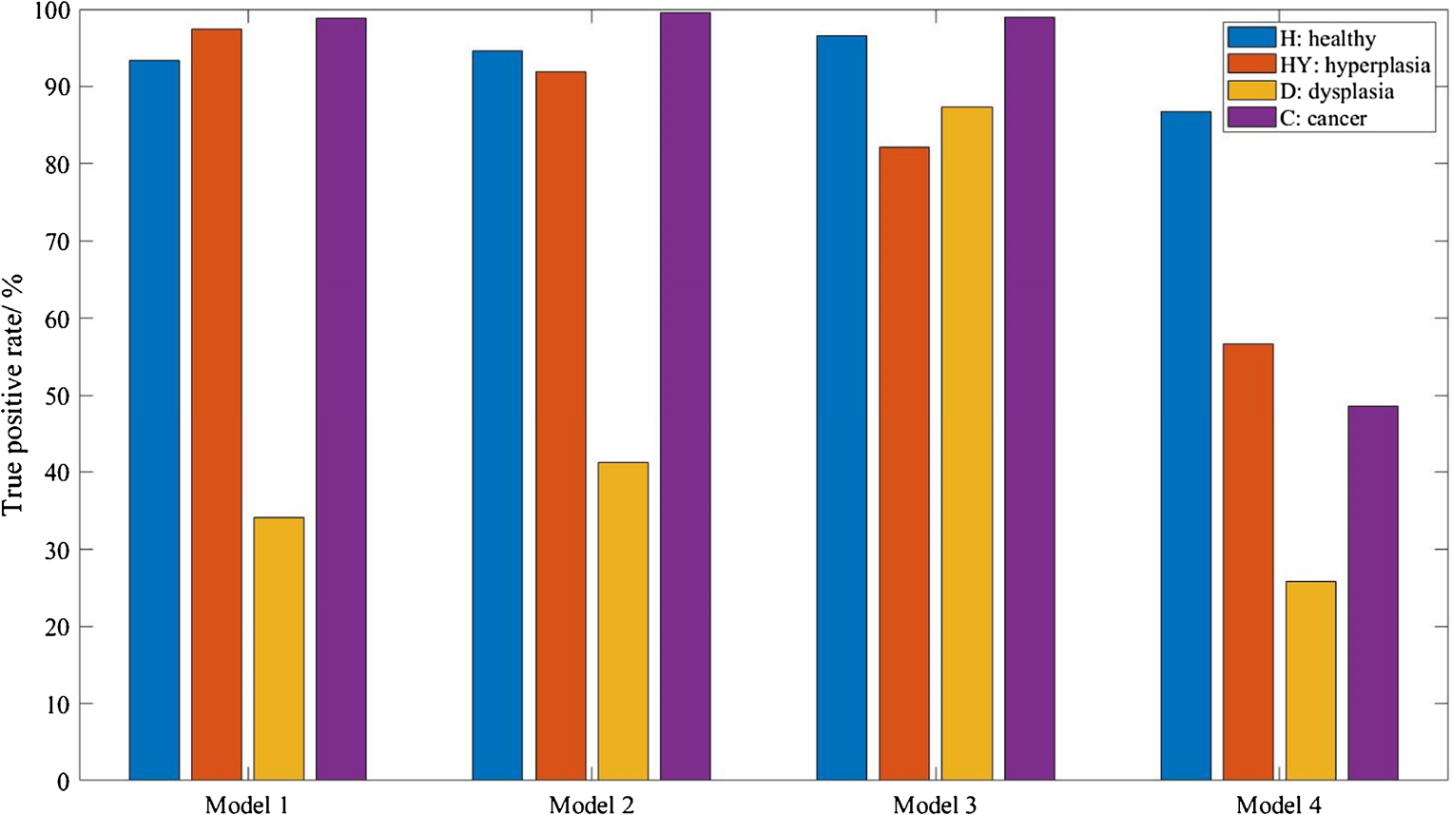
The bar chart shows the prediction accuracy of different stages of colon disease within each model of cluster 2 (high lipid absorbance area) for measurement without lens

On the other hand, model 4 gives the lowest prediction accuracy; this infers that lipid spectral region (or high wavenumber region) alone is not reliable for supervised training of the classification model in the study of colon biopsy. Nevertheless, the possibility of classifying between normal and cancer state of a biopsy, without classification of the stages of disease, based solely on the lipid region is not ruled out. For example, the study by Pilling et al. for different types of cancer (breast cancer) and using different substrates that rely on high wavenumber spectral range alone allows for rapid discrimination between normal epithelium, malignant epithelium, normal stroma, and cancer-associated stroma of breast biopsies with classification accuracy as high as 95% [37]. However, the categorisation of the different stages of breast cancer was not shown in their study. The breakdown of the true positive rates (or true negative rates for non-healthy tissue) of each cancer grade (measurement without additional lens) is shown for all models of cluster 2 in Fig. 8.

From these results, it is apparent that healthy and malignant tissues are easily distinguished from other stages of the disease, whereas dysplastic tissue is often misclassified as hyperplasia, if the correct spectral range is not implemented. Hyperplastic and dysplastic tissues rely heavily on differences within 1500–1000 cm^−1^, possibly from the change in concentration of the nucleic acid and carbohydrates in the tissues [13], and can be classified at a high accuracy when only the fingerprint region is used. Hyperplasia and dysplasia exhibit very similar spectral pattern above 1500 cm^−1^; hence, they are best differentiated from each other when the amide and lipid bands, which have higher absorbance and would dominate over the nucleic acid bands when no vector normalisation is carried out, are eliminated from the training dataset (model 3). The results from supervised learning give a significant insight into assessing the spectral biomarkers of colon cancer.

**Fig. 9:**
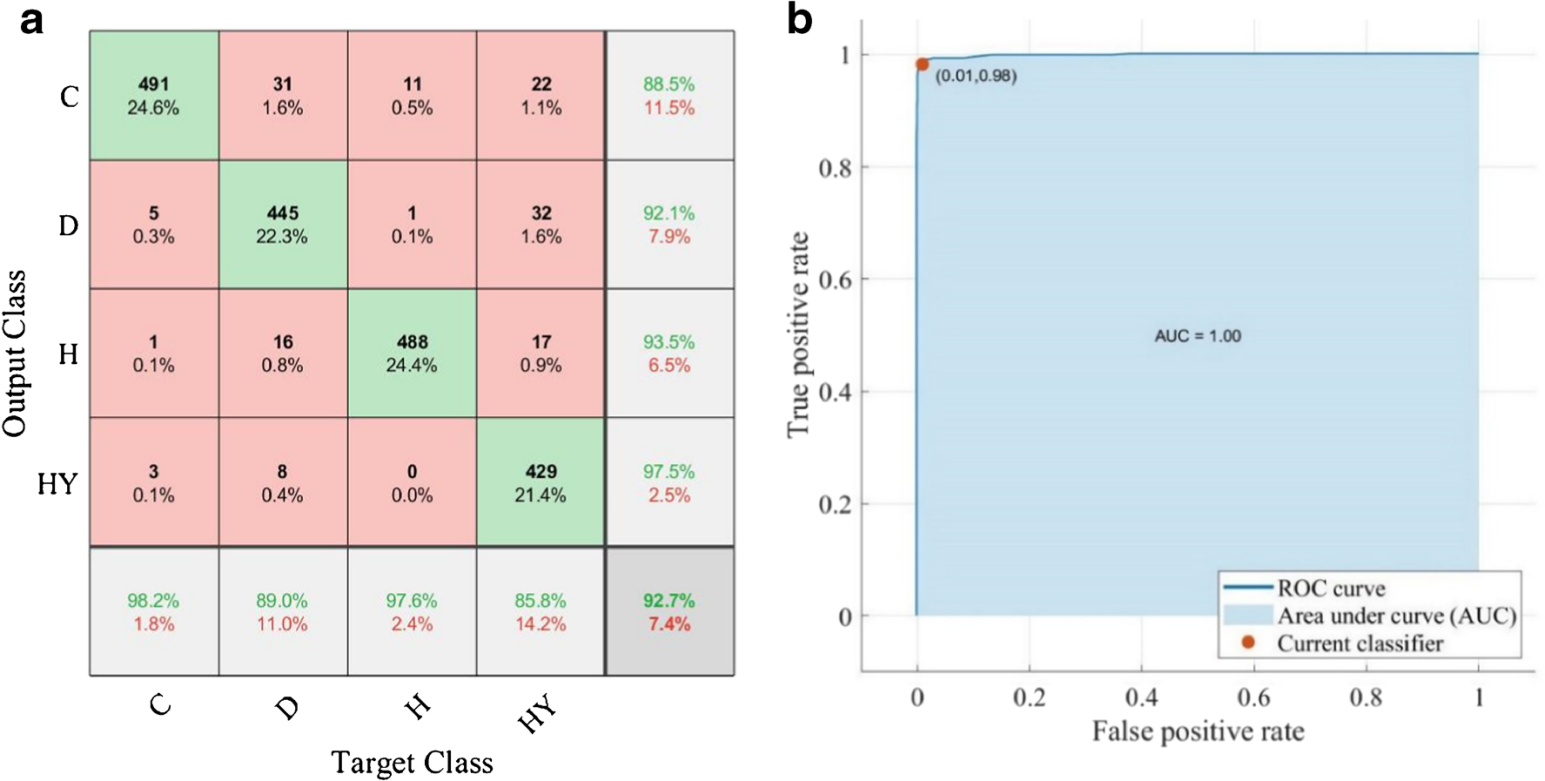
**a** The confusion matrix plot (MATLAB R2018b) shows the best result that can be obtained from the fingerprint region of the spectral data with model 3 (C, cancer; D, dysplasia; H, healthy; HY, hyperplasia). The rows show the predicted class and the columns represent the true class. The diagonal cells correspond to correctly classified observations, whilst the off-diagonal cells correspond to observations that are incorrectly classified. Both the number of observations and the percentage of the total number of observations are shown in each cell. The column on the right of the plot shows the percentages of all the examples predicted to belong to each class that are correctly and incorrectly classified. The row at the bottom of the plot shows the percentages of all the examples belonging to each class that are correctly and incorrectly classified. Overall accuracy of the prediction of the classifier model is given in the cell in the bottom right of the plot. **b** The ROC curve illustrates the diagnostic ability of the classifier system. The area under the curve (AUC) is ~ 1.0, which corresponds to a perfect classifier for the data in this study to distinguish between diseases

It is important to note that a high intra-model prediction (prediction within the training model without test model) does not warrant a high inter-model prediction (prediction with the test model). In this case, inter-model prediction is employed as a better and more reliable guide to verify the efficiency of the machine learning and should be carried out where possible. The stability of the training model is confirmed by decreasing the ratio of the size of training to test models from 1:1 to 1:6; the error in the prediction accuracy is a mere ± 2.0%. The optimum variance of PCA for the training of the model is found to be 99%; ca. 20% of the second derivative data within the fingerprint region contains useful information for data classification (see ESM Fig. S1 for results tested with variance of PCA retained ranging from 87 to 100%). The final best results of the prediction model summarised in a confusion matrix plot [38] and the corresponding receiving operator characteristic (ROC) curve of the random forest classifier are presented in Fig. 9.

The findings are reinforced by comparing the prediction outcome with that obtained from spectral data after correction with RMieS algorithm (and without the correcting lens). The performance of the fingerprint region with amide bands after RMieS correction shows significant improvement in the prediction accuracy compared with the second derivative data before correction (from 81 to 91% prediction accuracy), despite being slightly lower than that of the fingerprint region alone, due to the correction of the amide I band (92%). Correction with the RMieS algorithm is computational whilst correction with the added lens is a practical optical approach; thus, as expected, the RMieS algorithm provides a more precise solution which indeed yields a better overall prediction accuracy. The confusion matrices for both cases are provided in ESM Fig. S2 and Fig. S3 respectively. In this study, both correcting lens and RMieS correction are shown to be useful at correcting the scattering effect on amide I band but might not be necessary if classification of the stages of the colon adenocarcinoma via machine learning technique is the main objective as the training model without any correction for Mie scattering is sufficient to yield accuracy comparable with that after correction.

## Conclusion and outlook

Spectral data of colon biopsies obtained with a correcting lens for FTIR imaging show a significant reduction in spectral aberrations due to inhibiting Mie scattering, as was shown in our studies with other types of cancer tissues. Optical modification of the FTIR spectroscopic imaging with a CaF_2_ correcting lens has the advantage that the Mie scattering correction algorithm does not need to be performed. However, for this study the correction effect was not as good compared to the computational method. Here, we report the insignificance of the role of amide I band in machine learning for the first time. Importantly, the findings show that the disease states can be distinguished without resorting to the correction of Mie scattering effect. By using K-means clustering and RF classifier with PCA reduction, our work has demonstrated that optimisation of the training model by refining the selected range of FTIR spectral data can alter the prediction outcome.

The novelty of this work showed that the best prediction outcome for the studied colon biopsy samples were obtained when unsupervised learning of the C-H stretching bands is coupled with supervised learning of the spectral region between 1500-1000 cm^-1^. Hence, whilst the C-H stretching region is useful for intra-tissue segmentation, only the spectral range of 1500–1000 cm^−1^ is important for supervised machine learning. The amide I band can be excluded from data analysis altogether, as evidenced in the Gini indices obtained in this work. In addition, reliance on the C-H stretching spectral region (3000–2800 cm^−1^) alone in supervised learning gives the worst prediction. This exploratory study involving a manageable number of datasets successfully highlights the extraction of the most meaningful parts of the spectral data, which sets a framework for further validation of the predictive ability of a more sophisticated deep learning model in future work.

To summarise, further application of this method to an unknown colon biopsy sample is straightforward and potentially fully automated with simple programming. Initial K-means clustering (with the number of clusters set to two) on the C-H stretching bands alone will pick up regions of high lipid absorbance which will subsequently be fed into the already trained RF model that predicts the outcome of the malignancy stage of the specimen. The findings, though significant, are limited to FTIR spectroscopic imaging of the colon biopsy. Furthermore, Mie scattering is more pronounced in single-cell imaging than tissue; the results of this study are strictly limited to differentiation of disease progression in colon tissue specimens.

## Electronic supplementary material

ESM 1 (PDF 516 kb)
