# Supplementary material for: Fourier transform infrared spectroscopic imaging of colon tissues: evaluating the significance of amide I and C–H stretching bands in diagnostic applications with machine learning
Source: Anal Bioanal Chem. 2019 Aug 16;411(26):6969–81. doi: 10.1007/s00216-019-02069-6 (PMC6834539; doi:10.1007/s00216-019-02069-6)
Supplement: Supplementary file 1 — (PDF 516 kb) [file 216_2019_2069_MOESM1_ESM.pdf]

Analytical and Bioanalytical Chemistry

Electronic Supplementary Material

Fourier transform infrared spectroscopic imaging of colon tissues: evaluating the significance of amide I and C-H stretching bands in diagnostic application with machine learning

Cai Li Song, Martha Z. Vardaki, Robert D. Goldin, Sergei G. Kazarian

Table S1 Parameters of RMies – EMSC algorithm used in Matlab to correct for Mie scattering effect

Number of iterations	10
Number of PCs used	8
Lower range for scattering particle diameter / µm	2
Upper range for scattering particle diameter / µm	8
Lower range for average refractive index	1.1
Upper range for average refractive index	1.5
Reference spectrum	Matrigel

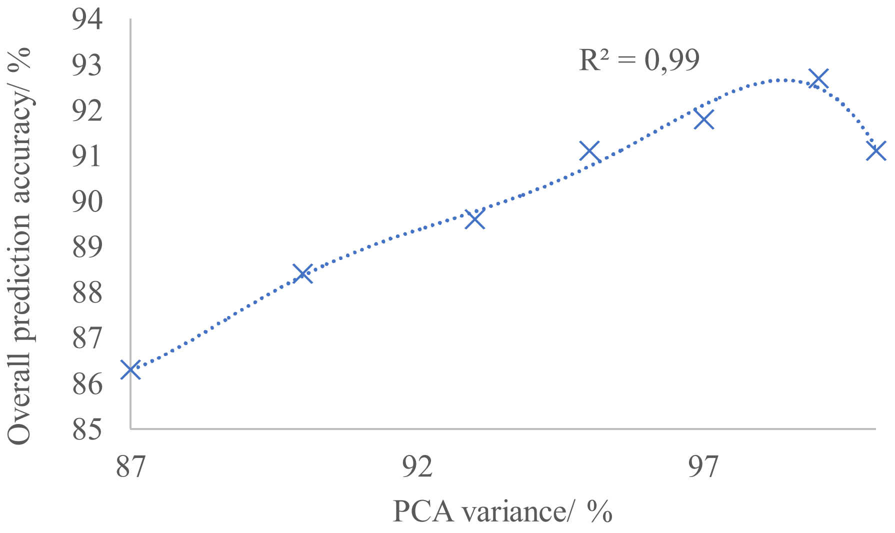

Fig. S1 A plot of overall prediction accuracy of RF classifier of the same range (within fingerprint region only) for PCA with variance ranging from 87% to 100%

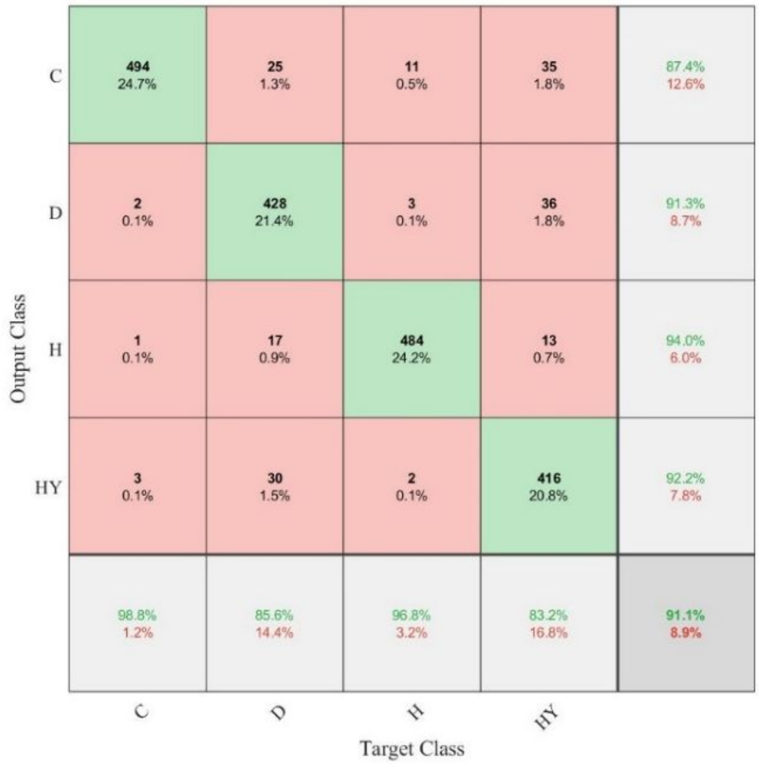

Fig. S2 The confusion matrix plot of the prediction outcome trained with the fingerprint and amide regions of the spectral data after correction with RMieS algorithm

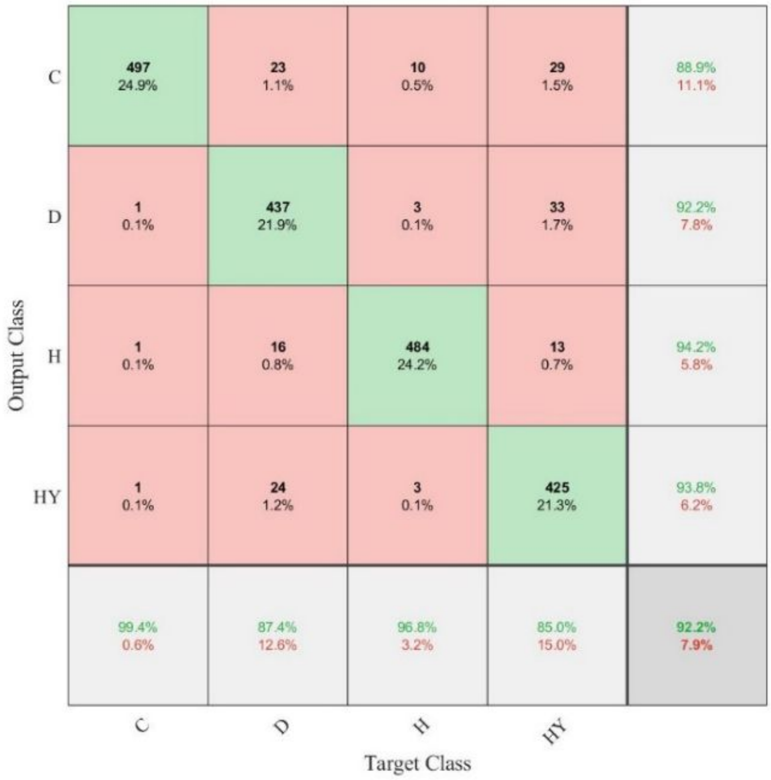

Fig. S3 The confusion matrix plot of the classifier trained with spectral bands of the data within the fingerprint region only after correction with RMieS algorithm

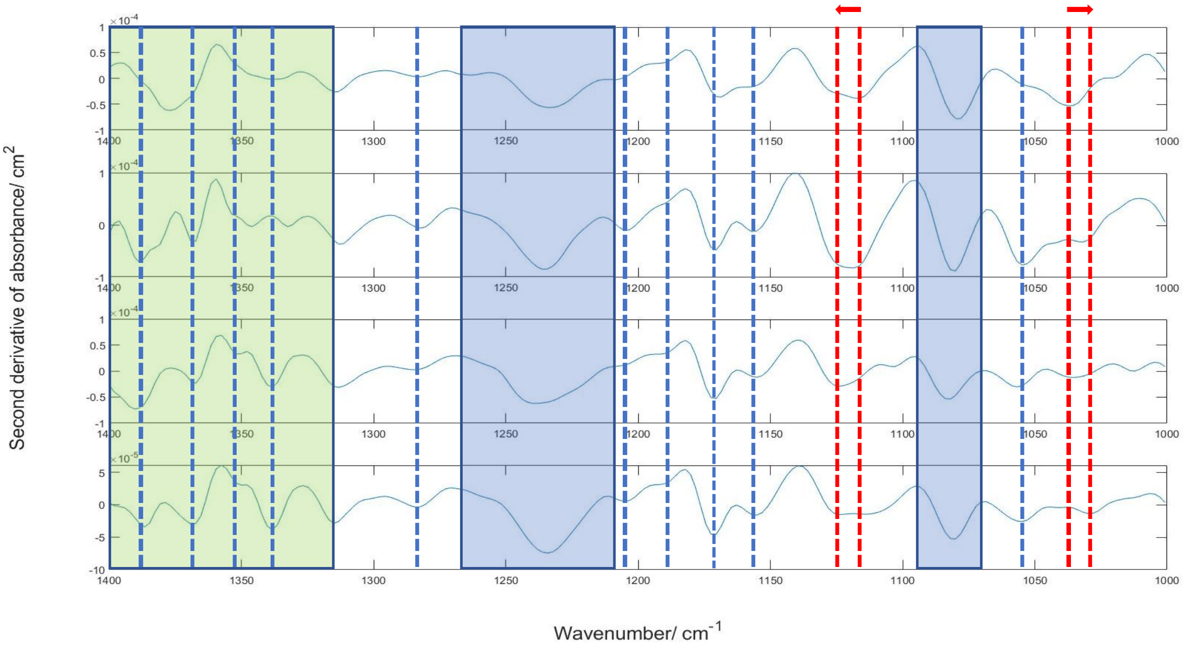

Fig. S4 The second derivative spectra of colon biopsy tissue (from top to bottom: healthy, hyperplasia, dysplasia, and cancer) within the spectral range of 1400 – 1000 cm^{-1} by taking average of all pixels of high lipid absorbance (high lipid cluster classified via k-means clustering technique after water vapour subtraction). The red dotted lines show the shift in spectral band as colon cancer progresses, whilst the blue dotted lines denote the peak where only slight change is detected in the intensity of the trough is observed. The blue regions show the spectral ranges where significant changes in intensity are observed. On the other hand, the green region denotes the spectral range susceptible to minor interference of the water vapour peaks. The comparison of the second derivative spectra in this region is compared before and after water vapour subtraction in Fig S5. The details of the spectral observation are tabulated in Table S2 below

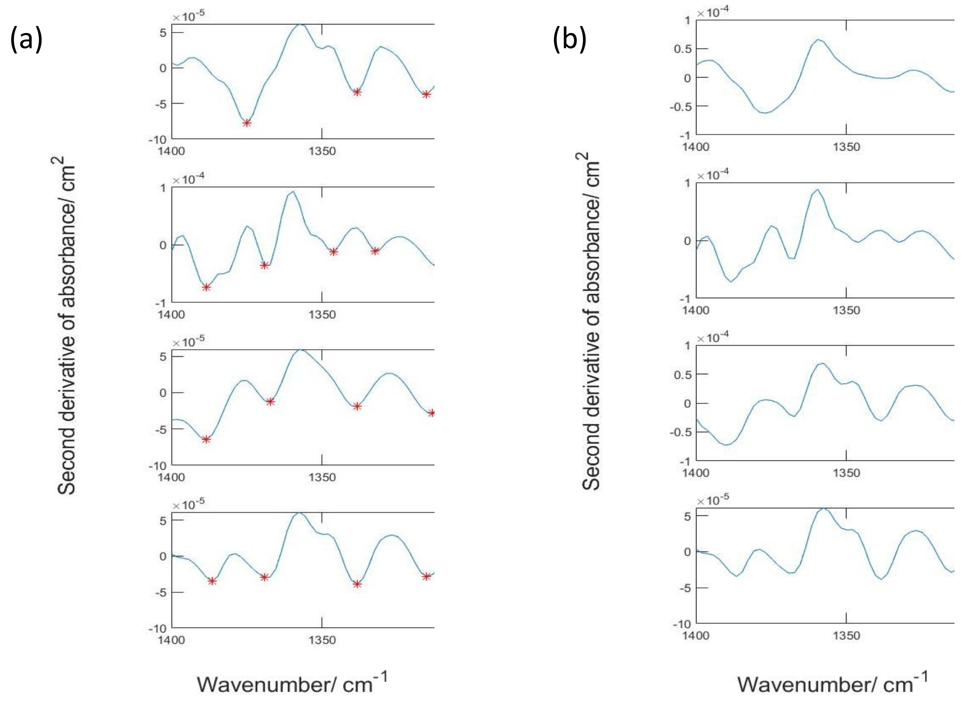

Fig. S5 Second derivative spectra in the range of 1400 – 1325 cm^{-1} before (a) and after (b) water vapour subtraction. The contribution of water vapour is very little in this study, so elimination of water vapour via water subtraction method does not necessarily improve the performance of the random forest predictive model, as discussed in the main article

Table S2 Differences in second derivative spectra with the increase in progression of colon cancer (from healthy to hyperplasia, followed by dysplasia and lastly cancer). Band assignment is taken from [1]

Wavenumber/ cm^{-1}	Band assignment	Observation in trough intensity	Observation in wavenumber shift
~ 1037	C-C, CH_2OH, C-O stretching coupled with C-O bending	-	Shift of band to lower wavenumber at 1030 cm^{-1}
~ 1050	C-O stretching coupled with C-O bending of the C-OH of carbohydrates; Glycogen	Insignificant in healthy tissue, significant in diseased tissues	-
~ 1080	Symmetric phosphate PO_2^- stretching; Collagen & phosphodiester groups of nucleic acids	Decrease	-
~ 1117	C-O stretching vibration of C-OH group of ribose (RNA)	-	Shift of band to higher wavenumber at 1124 cm^{-1}
~ 1155	C-O stretching vibration	Insignificant in healthy tissue, significant in diseased tissues	-
~ 1171	CO-O-C asymmetric stretching		
~ 1190	Deoxyribose	-	-
~ 1205	Amide III; Collagen	-	-
~ 1235	Composed of amide III as well as phosphate vibration of nucleic acids	Increase	-
~ 1263	PO_2^- asymmetric (phosphate I)	Significant only in healthy tissue	-
~ 1282	Amide III; Collagen	-	-
~ 1315	Amide III	Increase	-
~ 1338, 1352, 1367, 1386	CH_2 wagging; Stretching C-O, deformation C-H, deformation N-H	-	-

References:

1. Movasaghi, Z., Rehman, S. & ur Rehman, D. I. Fourier Transform Infrared (FTIR) Spectroscopy of Biological Tissues. *Applied Spectroscopy Reviews* **43**, 134–179 (2008).
